# Curling Up with a Story: An Interview with Sean Carroll

**DOI:** 10.1371/journal.pgen.1000229

**Published:** 2008-10-31

**Authors:** Jane Gitschier

**Affiliations:** Departments of Medicine and Pediatrics, Institute for Human Genetics, University of California San Francisco, San Francisco, California, United States of America

To meet Sean Carroll on his home turf in the early spring of Wisconsin is like encountering a bear cuddled up in his lair, waiting out the cold winter. I burrowed into the softly lit cave of small offices, with stalactites of yellow post-its dripping from every imaginable surface. Tiptoeing over misaligned stacks of books and reprints, I had to resist the urge to pick up one of the worn works, settle into a corner, and join in the reverie.

**Image 1:**
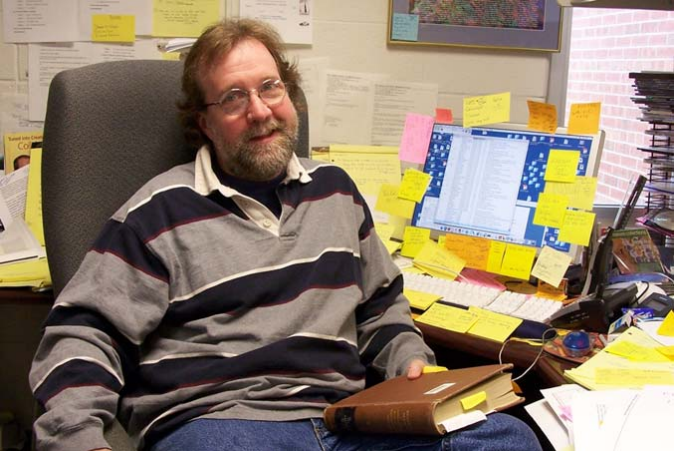
Sean Carroll.

Carroll (Image 1) is an expert in the field known as “evo devo,” an amalgam of developmental molecular biology as applied to the workings of animal evolution. Following his initial work with *fushi tarazu* (*ftz*)—one of the segmentation genes in the *Antennapedia* complex of *Drosophila*—he has been instrumental in elaborating the developmental regulation and interaction of a variety of genes, at first in the developing embryo, and later in the genesis of leg and wing appendages. A chance encounter fueled his long-standing interest in evolution and prompted him to re-tool his lab for the study of butterfly wing development; comparison between the two species led to groundbreaking insights into the subtle evolutionary changes that can give rise to spectacularly different appearances.

Carroll now leads a double life, and what captured my attention was his new-found voice as a writer about evolution, with three books already in print and, as I learned during the interview, two more ready for publication in 2009. We got the ball rolling by recalling how we had been introduced in Boulder, Colorado, while he was still a post-doc with Matt Scott, and I began by asking him about that period of time.

**Gitschier:** What took you to Matt's lab?

**Carroll:** Reading as a graduate student. I was an immunology graduate student at Tufts Medical School. I was even thinking that the evolution of the immune system was something to work on in the long term. But in those days, it took a lot longer to run gels, and you had time to read! So I read a lot, and I made use of the Red and Green Lines, getting around to all the schools in Boston. I went to seminars routinely at Harvard Cambridge, Harvard Med, MIT, and Tufts. And I went far afield, often, if it interested me.

**Gitschier:** What kinds of things did you read?

**Carroll:** All sorts of things—general science, general biology. Books by Stephen Jay Gould or his *Natural History* columns. History of science. Intense periods of science—atomic physics or cracking the genetic code. I had a strong appetite for that.

I had a growing awareness of issues and questions in evolution. At the time [early 1980s], punctuated equilibrium was a topic being discussed around Boston. And I thought a lot of this debate was about the evolution of form, about how quickly things could happen, and about the genetics of that. I realized you really couldn't have that debate without knowledge of what the genetics of form really were and without understanding how things were really built.

And that persuaded me that the next big step in evolutionary science in that vein was going to require an understanding of the genetics of animal development.

**Gitschier:** That was so specific!

**Carroll:** It was a distillation of a lot of cross-currents.

I looked around at what was going on. I came across two papers—one was the classic Ed Lewis review in 1978 on homeotic genes and the second was in 1980 by Nusslein-Volhard and Wieschaus, which is the report of the big screen in flies for zygotic mutants.

There were some whispers that things were starting to be understood molecularly, and that led me to the small group of labs that were working on fly developmental genes. One of those new labs was Welcome Bender's at Harvard. He said that he wasn't taking any more people, but he told me about Matt, who was wrapping up his post-doc work in Indiana with Thom Kaufman. I had some familiarity with Boulder, Colorado, and I thought: couldn't be the worst thing in the world to do post-doc in Boulder and work on these genes!

The work Matt had done as a post-doc essentially set the buffet. He walked through the whole *Antennapedia* complex but had not had time to work on any individual genes—how they were encoded, expressed, regulated.

So when I got to Boulder, it was open season on these genes.

**Gitschier:** Were you the first person in Matt's lab?

**Carroll:** Allen Laughon and I were there for day one in Boulder. Allen came from Ray Gesteland's [lab] in Utah. We took over a lab from a microbiologist, and Boulder hadn't bothered to clean it. So Al and I spent the first few days emptying reagents from old bottles and re-filling them with new ones.

We had a DNA map of the *Antennapedia* complex. The whole region, a few hundred kb, was cloned. Breakpoints of *scr* [sex-combs reduced] and *ftz* mutants were mapped.

I had an immunochemistry background, so I had a lot of experience in producing, purifying, and using antibodies. So I had something to bring to the table, but I had never worked on flies. The idea was to localize these gene products during development.

**Gitschier:** In your first book, you talk about this frustration of 1.5 years of work, and then coming out of the darkroom—

**Carroll:** Today is the anniversary—April 11, 1985—I even know the day!

**Gitschier:** I'm so honored to be here! So, what was the experiment? You were trying to localize *ftz* protein in the developing fly embryo.

**Carroll:** Well, it was really hard to know the path to take. In vitro, you could characterize an antibody and know that it was reacting with an antigen. But the methods for localizing antigens in embryos and imaginal discs were still emerging. A couple other labs were having some success. There was antibody to *Ubx* [*Ultrabithorax*] by that time. Tim Karr was working on fly embryos and had some protocols.

There was a lot of groping—a lot of lore about what vectors to use, β*-gal* fusion products, producing enough antigen, stability problems, purifying the antibody, how to permeabilize the embryo.

You didn't know if there was going to be enough antigen to see! I remember that was a criticism with Matt's grant: how do you even know there is enough protein to detect?

**Gitschier:** Well, you don't know!

**Carroll:** You don't know, and that's why we call it “*research*.”

The ultimate test was incubating the embryos with antibody and fluorescent secondary antibody and seeing! I don't know how many times that experiment failed in my hands. I devised a different way of purifying the antibodies in larger quantities, in bigger batches, in cleaning them up. I remember thinking, “I can't think of any better way to do this!” I was a year and a half into this, and I wasn't sure that I had any more tricks up my sleeve.

But then—it worked! It was early evening, hitting the scope, and just seeing green stripes [fluorescein-conjugated secondary antibody reacting with the primary antibody revealing *ftz* antigen in seven nuclear stripes]. In whole mount, it was a gorgeous thing to see!

Up to the time, people were doing in situ hybridization to sections and then exposing to film, and you'd have to wait for these things to develop for days and days—then you'd see the silver grain [deposits]. Ernst Hafen in Walter Gehring's lab had caught a nice tangential section that gave them a lot of the stripes. So, stripes of *ftz* RNA had been seen.

But there was something beautiful about seeing the nuclear protein in seven stripes. And I was looking at a pot of embryos that were *all* striped.

**Gitschier:** That must have been thrilling!

**Carroll:** Matt was home for dinner, as I recall. He came back in. And there *was* drinking. OK, I was drinking; Matt wasn't drinking.

**Gitschier:** It's too bad the published article itself doesn't show the color.

**Carroll:** No, in those days the articles weren't in color. But we did have the cover in color [together with work from Steve DiNardo and Pat O'Farrell on *engrailed*]. It was really brutal to get color images, for color slides and color prints—the cameras were mounted on the scope—they weren't digital, so you'd have to leave the shutters open for 30 seconds to get these pictures—and of course you're bleaching the embryos as you did that. The black and white images you could develop yourself in the lab, but the color stuff you had to send out and wait days to get back.

What the *ftz* and *engrailed* antibodies allowed us to do was to work out regulatory hierarchies. You had a batch of 20 or so loci that affected a segmental pattern—the gap genes, the pair-rule genes, and the segmentation genes. You had all these phenotypes, but you didn't know who regulated whom. The antibodies gave us tools to work this out pretty quickly. Rather than waiting for silver grains and the fortunate section, you'd stain a pot of embryos from a cross of a zygotic mutant line and you've got hundreds of mutant embryos—you've got a clear picture of whether gene expression is or is not altered. And bang! These reagents just sped up the analysis of regulation in space. And the resolution was great—cell-by-cell patterns of gene regulation, tips you got from spatial relationships of expression. Resolution in fluorescence microscopy is superb.

I remember people saying this could never be worked out—you had all these genes working very closely in time and in spatial patterns. You had to work on little pieces of the network. And that got into how individual genes were regulated.

**Gitschier:** As a post-doc, were you able to read as much as you had as a grad student?

**Carroll:** No, it was a lot of writing—pipette in one hand, pen in the other. It's going to sound awkward, but from the moment we saw stripes, there was a lot of writing! And writing takes time.

**Gitschier:** Obviously now you are a very prolific writer.

**Carroll:** Yeah, I've been de-repressed.

**Gitschier:** So was this instinct to write under some kind of repression that you weren't aware of? Did you *know* you liked to write? Had you been writing poetry or fiction, or keeping a journal?

**Carroll:** No! The only thing was that I took a second major, in French Literature. When I went to Washington University as an undergrad, I had to take a French class. And I thought “One French class, I'll bear it,” but the professor was fantastic. And I took five more classes with him after that including a graduate course, reading Rousseau, etc. You had to write for that—15-page-long term papers! To write in a foreign language and to write analysis of literature—somehow that was calisthenics for the writing brain and the writing voice.

But writing for science journals—there is a certain amount of DRYNESS to it that is ENFORCED by RIDICULOUS pressure. Did I say that loud enough? But writing of scientific papers requires a lot of discipline, a lot of logic, organization, succinctness.

**Gitschier:** Who was this inspirational French teacher?

**Carroll:** James F. Jones, but he goes by Jimmy Jones. He is now president of Trinity College in Hartford, Connecticut.

Last year, I was giving a public lecture at the American Museum of Natural History in New York. And he hired a bus and he brought faculty and students to the lecture and took me out to dinner after the lecture on Broadway. Thirty years later!

That's the caliber of the people at Wash U, and what amazes me now, from the position I now sit in, was how they made themselves available to the undergrads. I now realize that I was a pain in the [neck] and they never said so!

**Gitschier:** OK, you were probably going to use the word “wing development” before we digressed.

**Carroll:** Yes—back to the appendages. So my brain was saying “OK, we're movin' out, we're still thinking evolution”—but by late 1980s, I still haven't done anything explicit yet about evolutionary biology. I'm still preparing with developmental biology—understanding how to make a fly before we start thinking about other things and how they're different.

My early fire was *diversity*! So I wanted to study other animal models that would allow us to exploit what we had learned in flies and pursue questions of how diversity arose.

Then, a critical thing happened. I visited Duke University and I met Fred Nijhout. Fred was interested in endocrinology and a lot of other things—he had discovered the organizing center for the eye-spots in the butterfly wing by classic transplantation experiments in the imaginal disc.

I was talking about bristle patterns on the adult fruit fly, and Fred said “Do you think any of these genes you're studying could draw these kinds of patterns [on the butterfly wing]?” And that was the right question. And I said, “Yeah I think they could, so let's go find out.”

I decided butterflies were the right model to start asking questions about divergence and diversity. Butterflies have large hind wings, whereas fruit flies' second set of wings is the haltere. The scales on the wings were different—they are modified bristles. Their geometric color patterns were something new. And butterfly caterpillars have pro-legs on their abdomens. So all these are differences with respect to the body plan. And we probed all those differences.

That was the switch into the evo part of the evo devo for me—and that kind of flew out of control!

**Gitschier:** Obviously fruit flies are a lab animal, but butterflies? How did you gear up for that?

**Carroll:** Fred had a colony going for a long time. He sent us the butterflies, the recipe for the food. We learned from Fred how to grow them, so we had a constant supply of eggs in all developmental stages.

We made cDNA libraries, developed tools for in situs of embryos, made antibodies. Wing discs of butterflies are a lot bigger than fruit flies', so this was tricky getting them to look really nice when we probed them.

We cloned all the homeotic genes, the wing-patterning genes, and that gave us our early results. We posed very simply binary questions, and we got answers that were visual and that anyone could understand when they saw them.

It was about 2 years of technical investment before we started to get cool results. For example, of all the genes we study, one was used in a novel way—*distaless*, in the development of the eye-spot. Because it was this ancient gene, used in building legs, and it had taken on this new role, it was a striking, and at the time, I think, the first evidence of any kind of using old genes to make new patterns.

And the other thing, which I wasn't prepared for, was—goodness! How people like butterflies! Some of the public press things started because of butterflies.

**Gitschier:** When I first read about the butterfly work, I thought, “This is probably a guy who captured and pinned down butterflies as a child.” But then I read somewhere that you were into snakes!

**Carroll:** Yes—but it was all about color patterns!

**Gitschier:** Well, the butterfly stuff was really pivotal for you.

**Carroll:** It drew talent to the lab.

**Gitschier:** And it gave you some opportunities to try your hand at writing some news and views.

**Carroll:** When either a lot of data are emerging or it is a confusing situation and there is a need to distill, I like that challenge. In 1990, just as there was a sense of how periodic patterns were made in the embryo, I wrote a review for *Cell* about stripes. It was coming out, from Mike Levine's work, that inter-stripes were being repressed and stripes patterns were being carved from a block of potential expression by repressing expression in the inter-stripes. That article was the first effort on my own to try to get somewhere new conceptually.

**Gitschier:** How long did that take you to write?

**Carroll:** Months—anything takes me months. I can't even write a postcard in under a week. The re-writing, the honing, trying to draw figures that are helpful.

Then I started doing that more often, especially with the evolutionary stuff. In 1994, I wrote something for a meeting contribution—the first modern evo devo meeting, in Edinburgh.

**Gitschier:** Who coined the term “evo devo”?

**Carroll:** Don't know. I don't actually even like the word.

**Gitschier:** But it's the title of your book!

**Carroll:** It's the *subtitle* [of *Endless Forms Most Beautiful*]—my publishers like “evo devo”. I'm ok with it now.

By 1995, there were some misconceptions about homeotic genes and there were some new data, so I wrote a review for *Nature* in 1995. For a *Nature* audience, you've got to be aiming for those general themes, themes that have a root in history, what people had said before and how data were weighing in on long-standing questions. It's not just a snapshot of the last morsel of research; it's got to have perspective.

The desire and the necessity to write things like that increased. In 1996, 1997, we had some information on the evolution of limbs—the deep origin of limbs and some interesting comparative data with respect to vertebrate limbs. Neil Shubin, a paleontologist, invited me to write something with him and Cliff Tabin on the origin and evolution of limbs, and wow, three heads are better than one!

Then post–human genome project, there started to be a lot of chatter about human evolution. But some of the things being said, I felt, were not well grounded in what we already knew from model animals.

**Gitschier:** Like what?

**Carroll:** Too much anticipation that coding changes in proteins would explain a lot of our differences. Because, from the viewpoint of evolution of morphology, that was not what we were finding. The evolution of the human form—brains, bipedalism, neural wiring—I was motivated to tackle this. Can we anticipate what human evolution is all about, based on what we know about model organisms?

So I wrote a review article on that. That got me up to speed on hominid paleontology. I met paleontologists, read their papers. Hominid paleontology frames the issues. You've got to know the time scale of human evolution. At that point I had enough familiarity with a swath of material to tackle a book.

**Gitschier:** So how did that book [*Endless Forms Most Beautiful*] get off the ground?

**Carroll:** The trigger was that I was at a meeting, strolling the booths, and a Norton editor grabbed me. From their intel, they had heard that evo devo was something important. And they said they wanted to do something.

**Gitschier:** And this was at the same time you were thinking of doing a book.

**Carroll:** I was being asked to give some talks to general audiences about evolution of form. It's interesting to try to convey something in 50 minutes, but it was a vapor. How would the audience hold onto this? So a book would be a natural resource that they could have to follow through on some of this. I had a lot of warm-up for evo devo, so it was easy to get the riff going.

But I didn't know how to do a trade book—I didn't have an outline in my mind. With the live interest of Norton, I got serious. So that was the first step to entering this world, and it is a very different world!

**Gitschier:** In what sense?

**Carroll:** You have to learn some of its practices.

**Gitschier:** Like what?

**Carroll:** I did book tours each time. Regional NPR and National NPR, print interviews, public speaking, bookstore signings, doing Science Friday, giving a talk at a museum.

**Gitschier:** Did you enjoy that?

**Carroll:** Interesting experience.

**Gitschier:** So you are just a writing machine now.

**Carroll:** Now, it's psychotic. This is unsustainable—physically. Writing at the pace I have, putting out these two books. On the one hand, it is great for my soul. It's an interesting and creative challenge and very personally satisfying to hold yourself up and feel you have tackled some of that challenge.

And, as you get older in this business, and things aren't happening with your own pipetmen, it is nice to deploy a skill set and have some work to show for *yourself*. I'm sure it's made me a better scientist, because I think through things. I'm sure it's made me a better teacher, to be able to explain these things.

It's interesting too, working on evolution right now, because we have in this country the re-emergence of the dark ages!

So now, through book tours, I know writers at the daily newspapers, the commentators and the hosts. And I'm happy to be available when they have a question. I feel that part of my job is just to assist the media, because that's where people are getting a lot of their information.

Your sphere changes, and your sense of responsibility changes. You write these books, and you say, who reads them? Well—biology teachers! They rely on this to keep up with the science—I've worked with state and national teaching associations, my kids' school district, college board advanced placement test. What an interesting community that is!

You talk about outreach—this is what it's all about, and guess what, folks—it takes *time*!

I have just finished the third trade book and a spinout—a student book. Writing that, in the pure sense, was great. I was wading through the rich lore of natural history and some of the greatest people who ever lived—who wouldn't enjoy that? And I get to retell their stories in my own way but at the same time we've got some kick-tail research going on! And I have talks to give and journals to edit, so it's tricky.

**Gitschier:** I thought you said this writing business wasn't sustainable!

**Carroll:** This is *it*!

**Gitschier:** Can I quote you?

**Carroll:** Yes.

The student book is called “Into the Jungle”—subtitle: “Great Adventures in the Search for Evolution.” The premise is that textbooks—let's speak in genetic terms—they are necessary but insufficient. They don't convey the process of how science really gets done, and they don't give you any sense of the personality of the individual and the human drama of discovery.

**Gitschier:** That's why I do interviews!

**Carroll:** Exactly why! You wouldn't want to study movies by reading a textbook on movies—you want to *see* the stories!

So I felt that one thing I could do to contribute to teaching evolutionary science in a better way was to change the format. The notion of the book is that if a student could sit down with stories, much as they would sit down with a book of short stories in English lit class, and enjoy the stories the same way they would enjoy fiction—the drama, the characters, the places—that they would have a different experience and that the science would come across by osmosis, not by pedagogical hammer.

The people who first went into the jungle in the search for the origins of species were really admirable people who did remarkable things: Wallace, who lost all his specimens in shipwreck and was in open sea for 10 days; Dubois, who decided to quit his medical career and went off to find “apeman” fossils in Indonesia and discovered *Homo erectus*! He threw the golden dart.

I feel if that a student curls up with these stories in 10 or 12 pages, they can't shake it off. It shows how serendipity plays a role, and how, even when you find something great, the world isn't always ready to recognize it.

Let me show you something [as he opens a large old book]. This is what I get to read when I research a story. In the early 1920s Roy Chapman Andrews went across the Gobi dessert in search of ancient hominids—didn't find a one—but he went out in a fleet of Dodge cars with a camel caravan and this is the account of those expeditions. But instead, he found dinosaurs. These are the first dinosaur eggs.

**Gitschier:** How do you find all this stuff?

**Carroll:** I can't even tell you my process. I have a lot of help and a great library. So it's evolved. It's had its own little evolution.

